# The Association between Healthcare Satisfaction and Social Support and Stress, Depression, and Life Satisfaction in Female Caregivers: The Moderating Role of Dependence of a Sick Child

**DOI:** 10.3390/ijerph21091245

**Published:** 2024-09-20

**Authors:** Jadranka Pavić, Mateja Krznar, Snježana Čukljek, Biserka Sedić, Štefanija Ozimec Vulinec, Irena Kovačević

**Affiliations:** 1 Department of Nursing, University of Applied Health Sciences, 10000 Zagreb, Croatia; jadranka.pavic@zvu.hr (J.P.); biserka.sedic@zvu.hr (B.S.); stefanija.ozimec-vulinec@zvu.hr (Š.O.V.); irena.kovacevic@zvu.hr (I.K.); 2 Department of Nursing, Faculty of Health Studies, University of Rijeka, 51000 Rijeka, Croatia; 3 DEBRA, Croatian Epidermolysis Bullosa Association, 10000 Zagreb, Croatia; mateja@debra-croatia.com

**Keywords:** rare diseases, children, caregivers, social support, healthcare satisfaction

## Abstract

The caregivers of children suffering from rare diseases face numerous emotional, social, economic, organizational, and other difficulties, which can significantly impair their quality of life and mental health. Therefore, among other things, it is important to understand the factors which can influence psychosocial well-being. This research aimed to explore the association between healthcare satisfaction and social support and stress, depression, and life satisfaction in caregivers, with a moderating role of the ill child’s dependence on their caregiver. Methods: A cross-sectional study was conducted among 185 female caregivers of children with rare diseases. The data were analysed by using hierarchical regression analysis to examine the moderating effect of the child’s dependence. Results: Lower dependence of the child moderated the association between a higher level of healthcare satisfaction and reduced stress and a higher level of life satisfaction. Furthermore, lower child dependence moderated the association between a higher level of social support and a reduction in depression. In contrast, this association was absent in female caregivers with highly dependent children. On the other hand, the research confirmed that a higher level of social support led to stress reduction and increased life satisfaction in all respondents, regardless of the child’s dependence. Furthermore, the research confirmed that higher levels of healthcare satisfaction are associated with a reduction in depression in caregivers, regardless of the child’s dependence level. Conclusion: This research highlights the importance of providing adequate social support and high-quality healthcare in order to improve the psychosocial well-being of caregivers of children with rare diseases. Interventions to increase this support can reduce stress and depression and increase caregivers’ life satisfaction. Thus, future research should focus on the development and evaluation of specific interventions that support these factors.

## 1. Introduction

Rare diseases form a heterogeneous group of diseases and are mainly chronic, severe, exhausting, progressive, and related to a shorter life span. Despite being rare, rare diseases affect approximately 6% of the world’s population [1], i.e., more than 300–400 million people worldwide [2]. A total of 6000–8000 rare disease diagnoses have been identified, among which approximately 80% are genetic in origin, and 50–75% occur in early childhood [3,4].

Significant factors in the problems faced by persons suffering from rare diseases and their family members include the difficulties with diagnosis and access to treatment and medicines, as well as the lack of experience of healthcare professionals in discovering and treating these diseases [5].

Most rare diseases occur in childhood, so in most cases, the parents become the sick person’s primary caregivers. The shock of the initial diagnosis, the grieving process in accepting the disease, the fear of the progression of the disease, and the increased amount of care have a significant psychosocial and emotional impact on the family, and all difficulties are exacerbated by a lack of adequate community support [6,7,8,9].

In addition to obligations related to subsistence and daily activities, the family must face the demands of a family member’s illness. This means that new needs should be integrated into daily life and that life should be reorganized. Research on the experiences of patients and parents in everyday life and medical and psychosocial care shows a significant need for specific social support, including the support of healthcare professionals. In the absence of adequate support, families of patients with rare diseases report a lower quality of life due to post-traumatic stress disorder, loneliness, and more stress than parents of disease-free children [10,11,12].

The poorer functional status of the child is associated with multiple symptoms of the caregiver’s stress and depression [13,14]. Family caregivers of children dependent on the help of medical technology in caring for the child at home have high levels of depressive symptoms and relatively poor quality of life and family functioning. For example, research has shown a high prevalence of depression in caregivers who take care of children with long-term mechanical ventilation at home, in a vegetative state, or with severe disability [15,16].

More robust social support for the caregivers is important for their well-being [13]. Evidence shows that after the initial period of care at home, after discharge from the hospital, the support from family and friends tends to decrease over time [17]. In addition, a child’s serious disease often requires a significant amount of health and service support daily, which is difficult in certain situations. Patient satisfaction is a performance indicator showing the level of satisfaction with care provided by healthcare institutions and healthcare professionals [18]. Patient satisfaction with healthcare services is often evaluated when monitoring the quality of care, development, or evaluation of implemented interventions. Healthcare satisfaction indicators depend on a patient’s experiences, such as comfort, dignity during treatment, privacy, safety, autonomy in making decisions, and enabling personal preferences [19].

Families of children suffering from rare diseases receive poor attention from the political, scientific, and medical community. They face several obstacles, such as limited access to healthcare services, a lack of professional experience, and, generally, a lack of various types of support [20,21].

When taking care of their children suffering from rare diseases, parents face numerous challenges daily, such as changes in the way of daily functioning, and are burdened with various psychological, economic, emotional, and social difficulties [13,22]. As a result, parents often feel tired and experience poor physical health and social isolation [23]. The severity of a child’s condition and their dependence on the caregiver’s help affect the functioning of the family and their life satisfaction [14,15,24,25]. The most common difficulties expressed by the parents include physical weakness, cognitive and psychological difficulties, communication difficulties, and difficulties with physical activities [25].

### Research Aims and Hypotheses

This research aimed to examine the moderating role of the dependence of the ill child in the association between healthcare satisfaction and social support and stress, depression, and life satisfaction in female caregivers.

**H1.1.**  *A child’s dependence moderates a negative correlation between healthcare satisfaction and stress. On average, when the affected children are more dependent, the negative association between healthcare satisfaction and stress is statistically significantly higher.*

**H1.2.**  *A child’s dependence moderates a negative correlation between healthcare satisfaction and depression. On average, when the affected children are more dependent, the negative association between healthcare satisfaction and depression is statistically significantly higher.*

**H1.3.**  *A child’s dependence moderates a positive correlation between healthcare satisfaction and life satisfaction. On average, when the affected children are more dependent, the positive association between healthcare satisfaction and life satisfaction is statistically significantly higher.*

**H2.1.**  *A child’s dependence moderates a negative correlation between social support and stress. On average, when the affected children are more dependent, the negative association between social support and stress is statistically significantly higher.*

**H2.2.**  *A child’s dependence moderates a negative correlation between social support and depression. On average, when the affected children are more dependent, the negative association between social support and depression is statistically significantly higher.*

**H2.3.**  *A child’s dependence moderates a positive correlation between social support and life satisfaction. On average, when the affected children are more dependent, the positive correlation between social support and life satisfaction is statistically significantly higher.*

## 2. Materials and Methods

### 2.1. Study Design

This research was conducted in February and March 2024 on a convenient sample of parents of children suffering from rare diseases who are members of the Croatian Association for Rare Diseases. The ethics committee of the University of Applied Health Sciences gave consent to conduct the research (number: 251-379-10-24-02 of 22 January 2024). The research was anonymous, and it was conducted using Google Forms, which were sent to the 382 email addresses of the members of the Association. A total of 225 respondents filled in the questionnaire.

### 2.2. The Participants

Out of the total number of participants, 9.3% were men, out of which 8.4% were not married or in a relationship. Therefore, the analysis included only female caregivers who were married or in a relationship. There were 185 female caregivers.

Their average age was 46 [M = 45.98; SD = 10.99]. Since the distribution was highly asymmetrical, the participants were divided into three categories: up to the age of 36 [25.4%], age 37–55 [46.7%], and age 55 and above [27.7%]. When it comes to qualifications, due to a small number of participants in certain categories, some of the categories were merged. For example, the category of unskilled worker and completed secondary education contained 49.7% of the female caregivers, while the category of higher education, master’s degree, or PhD contained 50.3% of the participants. Regarding the place of residence, the following categories were merged: *island* and *village* [N = 29.2%] and *a smaller town* and *suburb* [N = 42.2], while the largest category was *a larger city* [N = 28.6%]. Regarding the number of children, 20.5% of female caregivers had one child, 50.8% had two children, and 28.6% had three or more children. The average age of the affected children was 10 [M = 10.23; SD = 5.70]. Due to the significant asymmetry in the distribution, the affected children were divided into three age categories: up to the age of 6 years [25.5%], 7–14 years [46.7%], and 14 years and older [27.7%].

### 2.3. The Instruments

#### 2.3.1. Sociodemographic Data Questionnaire

In the sociodemographic data questionnaire, the respondents answered questions regarding gender, age, qualifications, employment, place of residence, number of children in the family, and the age of the affected child.

#### 2.3.2. Questionnaire on the Child’s Dependence on the Help of Another Person

The questionnaire was constructed for research purposes and measured a child’s dependence on the help of another person in performing daily activities. It consisted of eight statements in which the participants evaluated the child’s dependence on the help of another person in the following areas: feeding, communication, personal hygiene, dressing, taking medication, going to the toilet, help with changing diapers in case of incontinence, and help with leisure activities. The answers were provided using a Likert scale from 1 to 5 (1—never, 5—all the time). An example of a statement is as follows: *“My child needs help with eating and drinking”.*

The Patient Satisfaction Questionnaire Short Form—(*PSQ-18*) [26]. This questionnaire was validated for use in different settings, and its internal consistency and reliability were confirmed. It consisted of 18 statements, and it evaluated seven dimensions of patient satisfaction directed towards doctors (general satisfaction, technical quality, interpersonal relationship, communication, financial aspects, time with the doctor, availability, and convenience). The participants evaluated every statement on a Likert scale from 1 to 5 (1—I completely agree; 5—I completely disagree). An example of a statement is as follows: *“Healthcare professionals sometimes ignore what I am saying.”* The total result on a scale is the average number of points on all statements, and a higher score suggests higher patient satisfaction. The internal reliability coefficient Cronbach alpha in this research is 91.

#### 2.3.3. MOS Social Support Survey [27]

A multidimensional scale of perceived social support (MSPSS) measures the perception of support from three sources: family, friends, and other important persons. The scale consists of 12 items, with 4 items for each subscale. The participants had to evaluate the statements regarding other persons’ support on a Likert scale: 1—I completely disagree, 6—I completely agree. An example of a statement is, “*There is a special person who is here when I need them.*” The total result is calculated by adding up all the answers. The score on the scale is calculated as the average number of points on all statements, and a higher score suggests greater social support. The internal reliability coefficient Cronbach alpha is 0.95.

Perceived Stress Scale (PSS) [28]. This scale represents a global measure of perceived stress in a person’s life. It consists of 10 statements about feelings and thoughts during the previous month. On a scale from 1—never to 5—very often, the participants must evaluate how often they felt overwhelmed or thought their life was out of control or unpredictable. An example is, *“During the previous month, how often did you feel that problems were piling up and that you were not able to overcome them?”* The total score on a scale is the average number of points on all statements, whereby a higher score represents a higher perceived stress of the participant. The internal reliability coefficient Cronbach alpha in this research is 0.89.

Depression Scale: *Center of Epidemiologic Studies Depression Scale, 10-item version*, CES-D—10), [29]. This is a short self-evaluation scale designed for measuring the self-evaluation of depression-related symptoms. The questionnaire consists of 10 statements that reflect the main aspects of depression: depressive mood, feelings of guilt and worthlessness, helplessness and hopelessness, psychomotor retardation, loss of appetite, and sleep disorder. On a scale of 0 to 3, the participants must evaluate symptoms in the past week (0—rarely or almost never, 1—a little, 2—sometimes, 3—all the time or almost all the time). An example is, “*During the previous week, I was worried about the things I don’t normally worry about.*” The possible range of results is 0–30. The total score on the scale is the average number of points on all statements, and a higher score suggests greater depression-related symptoms.

The internal reliability coefficient Cronbach alpha in this research is 89.

#### 2.3.4. The Satisfaction with Life Scale (SWLS) [30]

This scale consists of five statements that measure the cognitive evaluation of life satisfaction (an example is, *“My life is almost perfect in all aspects”.* The participants rate the extent to which they agree with a particular statement using a seven-point scale from 1 (*I completely disagree*) to 7 (*I completely agree*). The result on the scale is the average of the evaluations on all statements. A higher score suggests a higher level of life satisfaction. The internal reliability coefficient Cronbach alpha in this research is 75.

## 3. The Results

### Statistical Analyses Overview

Before the statistical analyses, the assumptions for their implementation were checked. All variables had indices of asymmetry and kurtosis within acceptable limits. A skewness and kurtosis value between −1 and +1 is considered excellent, while −2 to +2 is generally acceptable. Values beyond −2 and +2 suggest substantial non-normality. Thus, the data were processed using parametric methods [31].

In order to answer the problem of the moderating role of child dependence in the association between satisfaction and healthcare and social support on the one hand and stress, depression, and life satisfaction in female caregivers on the other, three moderation analyses were conducted. The independent variables were as follows: satisfaction with social care and social support. The dependent variables were as follows: stress, depression, and life satisfaction, and the child’s dependence was a moderating variable. The covariates were the aforementioned sociodemographic variables.

## 4. Descriptive Factors for the Examined Variables

In Table 1, it is evident that the participants rated the child’s dependence slightly above average, social support significantly above average, and satisfaction with social care slightly below average. Stress was evaluated as slightly above average, depression as average, and life satisfaction as slightly above average.

A child’s dependence is not related to any variable except weakly negatively with life satisfaction. Other variables are expected to have low or moderate correlations. Social support positively correlates with satisfaction with healthcare and life satisfaction and negatively with stress and depression. Satisfaction with healthcare negatively correlates with stress and depression and positively with life satisfaction. As expected, stress and depression have a high positive correlation with each other and a negative correlation with life satisfaction.

### 4.1. Moderation Analyses

#### 4.1.1. Moderating Role of Child’s Dependence in Association between Healthcare Satisfaction and Stress, Depression, and Life Satisfaction in Female Caregivers

In order to answer the problem of the moderating role of the child’s dependence in the association between healthcare satisfaction on the one hand and stress, depression, and life satisfaction in female caregivers on the other, three moderation analyses were conducted. The independent variable was social care satisfaction; the dependent variables were stress, depression, and life satisfaction; and the child’s dependence was a moderating variable. The covariants were the aforementioned sociodemographic variables.

##### Stress

Regarding the stress criterion variable, the interaction between child dependence and satisfaction with healthcare is significant (b = 0.18; t = 3.82, *p* < 0.001; 95% [CI 0.09, 0.27]). The association between healthcare satisfaction and female caregiver stress is moderated by the child’s dependence. The results of the moderation analysis are shown in Table 2.

The analysis of simple slopes revealed statistically significant effects of healthcare satisfaction in predicting stress in female caregivers of low-level dependent children (b = −0.48; t = −5.60; *p* < 0.001; 95% CI [−0.65, −0.31]) and moderately dependent children (b = −0.24; t = −3.81; *p* < 0.001; 95% CI [−0.37, −0.12]), but not in caregivers of highly dependent children (b = 0.00; t = −0.03; *p* > 0.05; 95% CI [−0.18, 0.18]).

**Figure 1:**
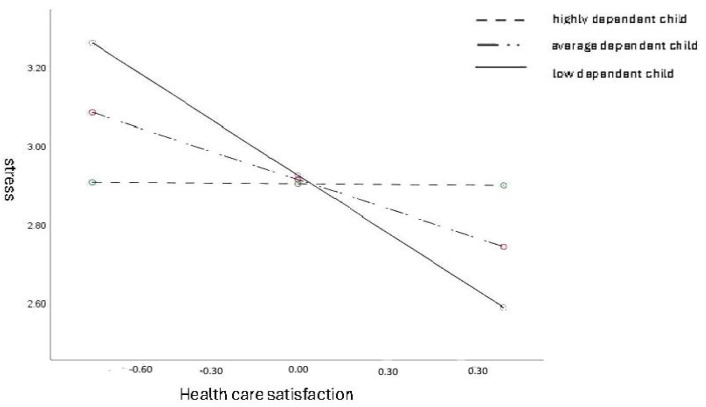
The child’s dependence as a moderator in the association between healthcare satisfaction and stress.

In the values of the regression coefficients and Figure 1, it is evident that the association between healthcare satisfaction and stress is the highest for female caregivers with a low-level dependent child (the steepest regression direction) and lower for those with a moderately dependent child. There is no such relationship in female caregivers with a highly dependent child. Those caregivers do not experience lower stress in the event of increased satisfaction with healthcare.

##### Depression

Regarding the depression criterion variable, the interaction between child dependence and healthcare satisfaction is not significant (b = 0.07; t = 1.65; *p* > 0.05; 95% CI [−0.01, 0.15]). The association between healthcare satisfaction and depression in female caregivers is not moderated by child dependence. The more satisfied the caregivers are with the quality of healthcare, the lower their level of depression is, regardless of the child’s degree of dependence.

##### Life Satisfaction

Regarding the life satisfaction criterion variable, the interaction between the child’s dependence and healthcare satisfaction is significant (b = −0.23; t = −2.64; *p* < 0.01; 95% CI [−0.09, −0.06]). The association between healthcare satisfaction and caregiver stress is moderated by child dependence. The results of the moderation analysis are shown in Table 3.

The analysis of simple inclinations slopes revealed statistically significant effects of healthcare satisfaction in predicting the life satisfaction of caregivers of low-level dependent children (b = 0.68; t = 4.41, *p* < 0.001; 95% CI [0.38, 0.99]) and moderately dependent children (b = 0.38; t = −3.36, *p* < 0.01; 95% CI [0.16, 0.61]), but not in caregivers of highly dependent children (b = 0.09; t = −0.52, *p* > 0.05; 95% CI [−0.24, 0.41]).

**Figure 2:**
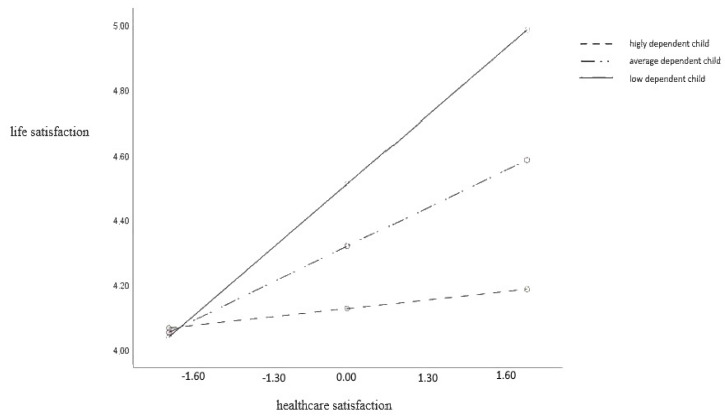
A child’s dependence as a moderator in the association between healthcare satisfaction and life satisfaction.

The values of the regression coefficients and Figure 2 show that the association between healthcare satisfaction and life satisfaction is the highest for caregivers with a low-level dependent child (the steepest regression direction) and lower for those with an average dependent child. There is no such association in caregivers with a highly dependent child. Those caregivers do not experience higher life satisfaction in the event of increased healthcare satisfaction.

#### 4.1.2. Moderating Role of Child’s Dependence in Association between Social Support and Stress, Depression, and Life Satisfaction in Female Caregivers

In order to answer the problem of the moderating role of child dependence in the association between social support on the one hand and stress, depression, and life satisfaction of caregivers on the other, three moderation analyses were conducted. The independent variable was social support; the dependent variables were stress, depression, and life satisfaction; and the child’s dependence was the moderating variable. The covariants were the aforementioned sociodemographic variables.

The moderating effect was obtained only for the depression criterion variable, while it was insignificant for stress and life satisfaction variables.

##### Stress

Regarding the depression criterion variable, the interaction between child dependence and social support is insignificant (b = 0.05, t = 1.82; *p* > 0.05; 95% CI [−0.004, 0.10]). The association between social support and stress in female caregivers is not moderated by the child’s dependence. Social support leads to reduced stress in female caregivers, regardless of the degree of the child’s dependence.

##### Depression

Regarding the depression criterion variable, the interaction between a child’s dependence and social support is significant (b = 0.05; t = 2.32, *p* < 0.05; 95% CI [0.01, 0.10]). The association between social support and depression in female caregivers is moderated by child dependence. The results of the moderation analysis are shown in Table 4.

The analysis of simple slopes revealed statistically significant effects of social support in predicting stress in female caregivers of low-level dependent children (b = −0.23; t = −5.01, *p* < 0.001; 95% CI [−0.32, −0.14]) and moderately dependent children (b = −0.17; t = −4.82, *p* < 0.001; 95% CI [−0.22, −0.09]), but not in female caregivers of highly dependent children (b = −08; t = −1.90, *p* > 0.05; 95% CI [−0.17, 0.00]).

**Figure 3:**
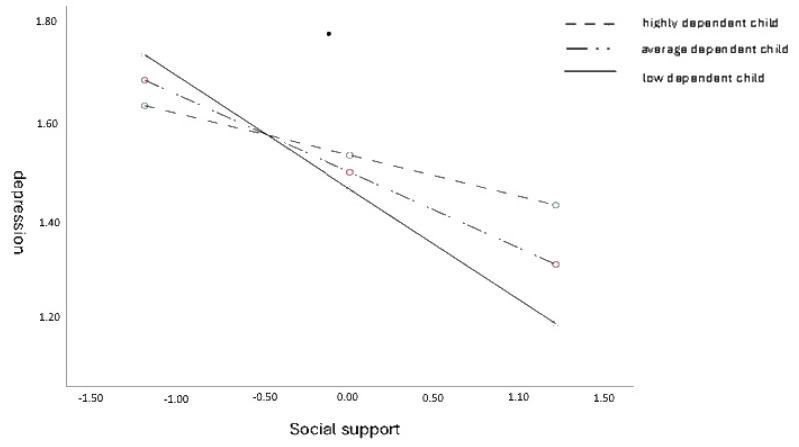
A child’s dependence as a moderator in the association between social support and female caregivers’ depression.

The values of the regression coefficients and Figure 3 show that the association between social support and depression is the highest for female caregivers with a low-level dependent child [the steepest regression direction], lower for those with a moderately dependent child, and the lowest for those with a highly dependent child.

##### Life Satisfaction

Regarding the life satisfaction criteria variable, the interaction between a child’s dependence and social support is insignificant (b = 0.04, SE = 0.04 t = 1.04, *p* > 0.05), which suggests that the association between social support and life satisfaction in female caregivers is not moderated by the child’s dependence. Social support positively correlates with life satisfaction, regardless of the degree of the child’s dependence.

## 5. Discussion

This research aimed to examine the moderating role of the child’s degree of dependence in the association between healthcare satisfaction and social support on the one hand and the life satisfaction, stress, and depression of the child’s caregivers on the other. It was assumed that the negative association between healthcare satisfaction and social support, on the one hand, and stress and depression, on the other, would be greater in caregivers who have a child with a high degree of dependence, as would the positive association with life satisfaction. The results partially confirmed the hypotheses.

### 5.1. The Moderating Role of the Degree of the Child’s Dependence in the Association between Healthcare Satisfaction, Stress, and Depression

Hypotheses H1.1, H1.2, and H1.3, on the significant moderating role of the child’s dependence in the association between healthcare satisfaction and stress, depression, and life satisfaction, were not confirmed. The child’s degree of dependence has a moderating role, but not for all variables and the expected direction.

#### 5.1.1. The Moderating Role of the Degree of the Child’s Dependence in the Association between Healthcare Satisfaction and Stress

Contrary to expectations, the association between healthcare satisfaction and life satisfaction and stress is the highest for female caregivers who have a low-level dependent child and slightly lower for those who have a moderately dependent child. For those caregivers, increased healthcare satisfaction leads to increased life satisfaction and stress reduction. There is no such association for female caregivers with a highly dependent child. Those caregivers do not experience higher life satisfaction or reduced stress in the event of increased healthcare satisfaction. Previous research has confirmed that healthcare satisfaction is associated with a higher level of life satisfaction and reduced stress among caregivers [32,33].

One of the explanations for our results lies in the education provided by healthcare professionals to caregivers of highly dependent children. Such children often face life-threatening situations and require continuous care by caregivers, so health professionals must fully train caregivers to take care of the child independently. For this reason, trained caregivers, as well as their competencies in providing care to a highly dependent child, which they have developed through experience, result in the caregivers achieving significant expertise and confidence in the care of their children [33,34,35,36]. Therefore, their healthcare satisfaction is not essential for stress reduction and overall life satisfaction. However, regardless of healthcare satisfaction, parents of highly dependent children have more burdens in their daily lives, which leads to more stress and reduced quality of life. These are burdensome factors in everyday functioning, such as the organization of everyday life, financial expenses, mutual relationships in the family, limitations in social life, etc. [37,38,39,40,41,42]. Furthermore, parents of highly dependent children have a significantly increased level of stress and a reduced quality of life due to daily difficulties and fear related to the complications of the child’s disease, the need for constant vigilance, and frequent medical examinations, which place significant time demands on the parents as well as needs in other dimensions of life that they cannot satisfy due to their child’s dependence [23,43,44,45,46,47].

Thus, although satisfactory health care is important, it may not significantly affect life satisfaction or reduce stress for caregivers of highly dependent children. Instead, other factors, such as practical support in daily tasks, emotional support, and social services that ease the burden of caregiving, play a more critical role in improving life satisfaction and reducing stress for these parents.

#### 5.1.2. The Moderating Role of the Degree of the Child’s Dependence in the Association between Healthcare Satisfaction and Depression

Furthermore, unlike the relationship between healthcare satisfaction, life satisfaction, and stress, the link between healthcare satisfaction and depression in female caregivers is not conditioned by the child’s level of dependency. Regardless of the child’s dependency level, greater healthcare satisfaction is consistently associated with lower levels of depression in caregivers. This suggests that the quality of healthcare contributes to caregivers’ mental well-being by reducing feelings of helplessness and emotional burden. Previous research has shown that parents of children with chronic illnesses often experience heightened levels of anxiety, insecurity, loss of self-esteem, negative emotions, fear related to the child’s illness, and depression [47,48]. These psychological challenges further underscore the importance of comprehensive healthcare support to improve caregivers’ mental health.

The burden of caregivers of children with severe, chronic conditions, such as muscular dystrophy, can have significant effects on mental health. Studies show that caregivers’ burdens are associated with depression [49,50], distress [46,51], anxiety, and loneliness [52]. When taking care of a child outside of a healthcare institution, parents have a complex role and assume several responsibilities and complex interventions, such as introducing a nasogastric tube [53], taking care of tracheostomy [54], anal dilation [55], etc. Unpleasant experiences, uneasiness, and suffering of the child lead to emotional difficulties for the parents. Therefore, the support of health personnel provided to caregivers is not only a positive practice, but also a form of emotional support [56].

Previous research confirms that healthcare satisfaction is associated with a reduction in depression in caregivers. Healthcare satisfaction can increase parents’ safety regarding the child’s treatment, as well as reduce helplessness, which is also related to depression [57]. Research has shown that continuous support by healthcare professionals, which is provided to the parents of children with severe and chronic illnesses, has significant effects on reducing the symptoms of burnout, depression, and anxiety in parents [58,59]. Our research has confirmed that healthcare satisfaction reduces depression in all female caregivers, regardless of the degree of the child’s dependence. Research has shown that in highly dependent children, high-quality healthcare cannot reduce stress due to daily problems. However, previous research suggests the importance of support from healthcare, which helps the caregivers in reducing helplessness, which is related to depression.

### 5.2. Moderating Role of the Degree of the Child’s Dependence in the Association between Social Support and Stress, Depression, and Life Satisfaction

This section examines the moderating role of the child’s dependence in the association between social support and the psychosocial outcomes (stress, depression, and life satisfaction) in caregivers. Contrary to our hypothesis, the child’s dependence did not significantly moderate the relationships between social support and stress or life satisfaction. However, a moderating effect was found for the depression variable, though not in the expected direction.

#### 5.2.1. Moderating Role of the Degree of the Child’s Dependence in the Association between Social Support and Stress

The association between social support and stress and life satisfaction in female caregivers is not moderated by the child’s dependence. Social support is negatively related to stress and positively related to life satisfaction, regardless of the degree of the child’s dependence. A family member’s disease can lead to a new life situation for other family members, in which the need to provide care redefines the relationship with other people, daily routine, and priorities. Parents of children with chronic health issues must adapt to new roles, reorganize their lives, and deal with increased demands in terms of care. Social support is an important predictor of life quality and life satisfaction in families with healthy and ill children [60]. It includes emotional, informational, and moral support, as well as socializing [60]. Emotional support is every piece of information that makes the person feel appreciated, understood, and accepted. Informational support refers to receiving information and assistance in defining, understanding, and dealing with the events. Material support includes receiving help for material costs and financial assistance. Finally, social support refers to a sense of belonging to the group, participating in it, and leisure activities with other people [60]. Social support can have a positive influence on adjusting to the disease and stress reduction in parents with children suffering from rare diseases [61]. Research conducted by Oers et al. showed that the parents of chronically ill children, especially mothers, reported a high level of anxiety and depression. Practical problems in daily lives and parenthood, together with stress, showed the strongest association with anxiety and depression. At the same time, the characteristics of a child related to the disease did not influence parents’ anxiety or depression [10]. Therefore, this research has also shown that social support is important to caregivers, regardless of disease severity and the child’s dependence.

#### 5.2.2. Moderating Role of the Degree of the Child’s Dependence in the Association between Life Satisfaction

Similarly, the hypothesis (H2.3) posited that the child’s dependence would enhance the positive correlation between social support and life satisfaction. However, our analysis revealed that the child’s dependence did not significantly influence this relationship. Social support positively correlated with life satisfaction for all caregivers, regardless of the child’s dependence. The lack of a moderating effect may be explained by the multidimensional nature of social support, which includes emotional, moral, and practical support. These aspects of support seem to provide a consistent benefit to caregivers’ overall life satisfaction, regardless of the specific caregiving challenges posed by the child’s condition. This finding underscores the overall importance of social support in improving caregivers’ quality of life [61]. Studies in the literature have shown a positive association between perceived social support and the psychological well-being of patients with chronic diseases [60,62,63] and that support in meeting basic needs is negatively correlated with psychological problems, including depression, and positively correlated with well-being and life satisfaction [64,65,66].

#### 5.2.3. Moderating Role of the Degree of the Child’s Dependence in the Association between Depression

When it comes to the depression variable, the degree of the child’s dependence has a moderating role. The negative association between social support and depression is the highest for female caregivers with a low-level dependent child (the steepest regression direction), lower for those with a moderately dependent child, and the lowest for those with a highly dependent child. These results can be explained by the fact that the caregivers of highly dependent children are focused on the basic everyday activities in terms of caring for their children, which last all day and are continuous, exhausting, and often accompanied by fear for the outcome of the child’s condition, which includes higher levels of anxiety and depression on which social support has less impact. For example, the caregivers of highly dependent children suffer from chronic sleep deprivation, which has numerous negative health implications. Furthermore, sources from the literature highlight the connection between poor sleep and increased levels of stress, anxiety, and depression [67,68,69]. Furthermore, parents of highly dependent children are constantly preoccupied and worried about their child’s condition [70,71], which is often related to the loss of perceived control because they have no choice in overcoming the complex care related to their child’s daily struggles. Such an everyday situation represents a high risk of depression [7,34,72,73,74].

Bearing these factors in mind, it is unsurprising that parents have little time to engage in their own meaningful activities, which, consequently, increases the risk of depression [75,76]. Even though social support is an important protective factor between parental stress and depression in caregivers of children with developmental difficulties [77], in our research, it is evident that caregivers of highly dependent children perceive support to a lower extent. Previous research has shown that caregivers who perceive a high level of social support are less likely to experience depression. The challenges faced by caregivers of children with chronic and rare diseases in particular need of social support include concern about the future, problems with their children’s jobs, social discrimination, and stigma, as well as financial difficulties [78]. When it comes to obstacles to their psychological well-being, caregivers of children with severe and chronic diseases reported difficulties with accessing social services and information, as well as with school and community integration during the upbringing of their child [78]. According to this study, which examined their needs, the caregivers of children with severe and chronic diseases also reported an increased need for support in treatment and education, vocational training for their children, and various programs, such as programs for enhancing social skills [79]. Based on the above results, it is evident that caregivers of children with severe and chronic diseases want to use all available resources to support their child’s psychophysical and social development, and, therefore, they perceive a required need for improving care. In highly dependent children, who depend on their caregivers’ help, there is a reduced possibility of using certain amenities. In contrast, less dependent children have more possibilities in terms of using social resources, including the selection of education and employment, and a higher possibility of using rehabilitation and social activities. For this reason, social support is more prominent and visible among caregivers of this population. Although social support is necessary for all caregivers, our research shows that it is the most significant negative moderator between a low- and less dependent child and female caregivers’ depression. Therefore, social support offers more possibilities in helping female caregivers of less dependent children because it can make daily life easier, mediate the realization of different needs, enable more independence, and reduce the risk of depression. On the other hand, for caregivers with highly dependent children, social support has fewer possibilities of helping in daily functioning because a highly dependent child has fewer opportunities for realization in different areas. The caregiver continuously participates 24/7in providing care, which reflects in their physical and mental health, i.e., a higher prevalence of depression among caregivers.

### 5.3. Limitations of the Study

This study has several limitations that should be taken into account when interpreting the results.

Limited number of male respondents: Due to the small number of male respondents and respondents who are not married or in a partnership, this study only analysed the answers of female respondents who are married or in a partnership. In future research, it would be useful to investigate whether there are differences concerning the gender and partner status of the respondents in relation to the examined variables in this research.

Cross-sectional design: The cross-sectional nature of this study restricts the ability to establish causal relationships between the variables. For instance, while associations between social support and psychological outcomes were observed, we cannot conclude whether greater social support leads to lower stress or if less stressed individuals perceive higher levels of support. Longitudinal studies would be better suited to examining how these relationships evolve over time.

Self-report measures: The reliance on self-report questionnaires introduces potential biases, such as social desirability or recall bias, which may affect the accuracy of the data. Caregivers may under- or overestimate their levels of stress, depression, or satisfaction with healthcare services. Future research could complement self-report data with objective measures such as clinical evaluations or reports from healthcare providers to offer a more balanced perspective.

Cultural and systemic differences: The study was conducted among caregivers of children with rare diseases in Croatia, and the results may not fully apply to caregivers in other cultural or healthcare contexts. Broader, more diverse samples in future studies would help to validate these findings in different environments. Furthermore, the study was conducted within the Croatian healthcare context, which may differ significantly from healthcare systems in other countries regarding resources, organization, and support for caregivers. These systemic differences could limit the applicability of the findings to other regions. Cross-cultural studies in different healthcare systems could provide valuable insights into how systemic factors influence caregivers’ well-being.

## 6. Conclusions

This research identified a significant association between social support, healthcare satisfaction, and psychosocial well-being in caregivers of children with rare diseases. Increasing the level of social support and improving the quality of healthcare can reduce stress and depression and increase caregivers’ life satisfaction. Future research should explore specific interventions that would further support these findings.

## Figures and Tables

**Table 1:** The basic descriptive parameters and correlations between the examined variables.

	1	2	3	4	5	6
1 Child’s dependence	-	−0.03	−0.00	0.02 **	0.08 **	−0.15 *
2 Social support		-	0.35 **	−0.33 **	−0.34 **	0.53 **
3 Satisfaction with healthcare			-	−0.29 **	−0.35 **	0.26 **
4 Stress					0.66 **	−0.47 **
5 Depression					-	−0.36 **
6 Life satisfaction						-
M	3.25	4.72	2.98	2.91	1.50	4.32
SD	1.32	1.20	0.70	0.63	0.55	1.13
Range of results	1–5	1–6	1.44–4.67	1.40–4.80	0.30–2.90	1.80–6.00
Skewness	−0.028	−1.156	0.137	0.077	−0.096	−0.479
Kurtosis	−1.388	0.522	−0.719	0.018	−0.656	−0.794

** *p* < 0.01; * *p* < 0.05.

**Table 2:** A presentation of the results of the moderation analysis for the stress criterion variable.

Variable	b	SE	t
Age	0.03	0.08	0.35
Qualifications	0.01	0.05	0.21
Place of residence	0.00	0.03	0.13
Number of children	−0.05	0.07	−0.70
Dependent child’s age	−0.09	0.07	−1.17
Child’s dependence	−0.02	0.04	−0.66
Healthcare dependence	−0.24	0.06	−3.38 ***
Interaction	0.18	0.05	3.82 ***

*Note.* Unstandardized coefficients are reported. *** *p* < 0.001.

**Table 3:** A presentation of the results of the moderation analysis for the life satisfaction criterion variable.

Variable	b	SE	t
Age	−0.09	0.13	−0.68
Qualifications	0.06	0.08	0.72
Place of residence	−0.09	0.05	−1.58
Number of children	0.17	0.12	1.41
Dependent child’s age	−0.09	0.13	−1.19
Child’s dependence	−0.15	0.06	−2.26
Healthcare dependence	0.38	0.11	3.36 **
Interaction	−0.23	0.09	−2.64 **

*Note.* Unstandardized coefficients are reported. ** *p* < 0.01.

**Table 4:** A presentation of the results of the moderation analysis for the depression criterion variable.

Variable	b	SE	t
Age	−0.01	0.06	−0.14
Qualifications	0.02	0.04	−0.06
Place of residence	0.00	0.03	−0.05
Number of children	−0.04	0.06	−0.15
Dependent child’s age	−0.02	0.07	−0.15
Child’s dependence	−0.16	0.03	0.82
Social support	0.03	0.03	−4.82 ***
Interaction	0.05	0.02	2.931 *

*Note.* Unstandardized coefficients are reported. *** *p* < 0.001 * *p* < 0.05.

## Data Availability

The datasets generated and analyzed for this study can be requested from the correspondent author. The data are not publicly available due to policy of institutions which gave ethical approval to the study.
